# Cooperation of Dnmt3a R878H with Nras G12D promotes leukemogenesis in knock-in mice: a pilot study

**DOI:** 10.1186/s12885-019-6207-y

**Published:** 2019-11-08

**Authors:** Xiaodong Shi, Ying Yang, Siqi Shang, Songfang Wu, Weina Zhang, Lijun Peng, Ting Huang, Ruihong Zhang, Ruibao Ren, Jianqing Mi, Yueying Wang

**Affiliations:** State Key Laboratory of Medical Genomics, Shanghai Institute of Hematology, Rui Jin Hospital, Shanghai Jiao Tong University School of Medicine, Shanghai, 200025 China

**Keywords:** Acute myeloid leukemia, DNMT3A mutation, Nras G12D, Myc activation

## Abstract

**Background:**

DNMT3A R882H, a frequent mutation in acute myeloid leukemia (AML), plays a critical role in malignant hematopoiesis. Recent findings suggest that *DNMT3A* mutant acts as a founder mutation and requires additional genetic events to induce full-blown AML. Here, we investigated the cooperation of mutant *DNMT3A* and *NRAS* in leukemogenesis by generating a double knock-in (DKI) mouse model harboring both Dnmt3a R878H and Nras G12D mutations.

**Methods:**

DKI mice with both Dnmt3a R878H and Nras G12D mutations were generated by crossing Dnmt3a R878H knock-in (KI) mice and Nras G12D KI mice. Routine blood test, flow cytometry analysis and morphological analysis were performed to determine disease phenotype. RNA-sequencing (RNA-seq), RT-PCR and Western blot were carried out to reveal the molecular mechanism.

**Results:**

The DKI mice developed a more aggressive AML with a significantly shortened lifespan and higher percentage of blast cells compared with KI mice expressing *Dnmt3a* or *Nras* mutation alone. RNA-seq analysis showed that *Dnmt3a* and *Nras* mutations collaboratively caused abnormal expression of a series of genes related to differentiation arrest and growth advantage. *Myc* transcription factor and its target genes related to proliferation and apoptosis were up-regulated, thus contributing to promote the process of leukemogenesis.

**Conclusion:**

This study showed that cooperation of *DNMT3A* mutation and *NRAS* mutation could promote the onset of AML by synergistically disturbing the transcriptional profiling with Myc pathway involvement in DKI mice.

## Background

DNA methytransferase 3A (DNMT3A), a member of DNA methytransferases family, is responsible for de novo DNA methylation, which is essential for genome regulation and development [1]. *DNMT3A* mutations have been identified in various hematologic malignancies, with frequencies of 20–25% in AML [2–6]. The hotspot mutation of DNMT3A in AML occurs at the residue Arginine 882 (R882) [2, 7]. Dnmt3a knock-out mice showed increased self-renewal and impaired differentiation of Hematopoietic stem cells (HSCs) [8–10]. Mouse models established through retroviral transduction system showed that DNMT3A R882H alone did not develop frank AML, but were susceptive to AML development upon acquisition of additional genetic mutations [11, 12]. Dnmt3a R878H which is homologous with human DNMT3A R882H, only induced moderate AML with an average of 20% immature cells in the bone marrow (BM) and a relatively long latency in the conditional knock-in mice model [13]. *DNMT3A* mutations were proved to play a key role in clonal hematopoiesis at premalignant stages [14, 15], whereas activated signaling genes including *RAS* and *FLT3* mutations occur in the subsequent process of malignant development [16]. Large scale sequencing of specimens from AML patients has discovered that *DNMT3A* mutations often coexist with other gene abnormalities, such as *FLT3*, *IDH1/2*, *NPM1* and *RAS* [2, 7, 17]. These findings suggest that abnormal *DNMT3A* acts as a founder mutation and requires additional genetic events to induce an aggressive full-blown AML.

*RAS* is mutated in ~ 25% of human cancers including AML and other myeloid malignancies [18, 19]. Mutations in *NRAS* have been identified in AML and coexist with DNMT3A mutations in a portion of AML patients [20, 21]. Mouse models showed that *NRAS* mutation alone was not sufficient to cause AML [22, 23]. Loss of Dnmt3a and endogenous Kras G12D cooperated to promote myeloid leukemogenesis in mice [24]. Besides, a previous report showed co-expression of DNMT3A R882H and NRAS G12D could induce mouse AML by using a retroviral transduction system, in which the expression of mutant DNMT3A and mutant NRAS were driven by a retroviral promoter instead of the endogenous promoter/enhancer [12, 25].. However, the cooperation of *DNMT3A* mutation with *NRAS* mutation under the control of endogenous promoters in inducing AML in mice which mimics human leukemic features and the underlying mechanism remains elusive. In this work, we report that Dnmt3a R878H cooperates with Nras G12D to develop frank AML by establishing a DKI mice model.

## Methods

### Generation of DKI mice

All mouse experiments were performed according to the guide of laboratory animal care and use standards, and were approved by the animal use committee of Shanghai Jiao Tong University. And all animals were maintained with sterilized water and food in the specific pathogen free circumstance in Research Center for Experimental Medicine at Rui Jin Hospital Affiliated to Shanghai Jiao Tong University School of Medicine. Mx1-Cre; Dnmt3a R878H KI C57 mouse model was established as described in our previous work [13]. Mx1-Cre; Nras G12D KI C57 mice were generously provided by Ren Lab from Shanghai Institute of Hematology. The Mx1-Cre; Dnmt3a^R878H/+^ KI mice were crossed with the Mx1-Cre; Nras G12D KI mice to obtain DKI mice harboring both Dnmt3a R878H and Nras G12D mutations. Cre expression was induced through intraperitoneal injection of 250 μg Polyinosinic-polycytidylic acid (pIpC) every other day for two times at 4 weeks old. The mice were monitored for leukemia development and sacrificed for phenotypic analysis 4 months after pIpC injection. The mice were sacrificed after the study. The method of euthanasia used to sacrifice the mice was cervical dislocation.

### Flow cytometric analysis

Peripheral blood (PB) was obtained from the tail vein of mice, red blood cells (RBCs) were lysed by RBC Lysis Buffer prior to staining. BM cells were flushed out from the tibias and femurs, and suspended in PBS buffer with 2% fetal bovine serum (FBS). The spleen cells were suspended as a single cell suspension in PBS buffer with 2% FBS. Cells were washed and resuspended in PBS buffer containing 1% FBS and subsequently stained with fluorochrome-conjugated antibodies (Biolegend) as following: PE anti-mouse Gr-1, APC anti-mouse Mac-1, BV421 anti-mouse B220, FITC anti-mouse CD3, FITC anti-mouse Lineage, PE anti-mouse Sca-1, APC anti-mouse c-Kit, APC-Cy7 anti-mouse CD48, BV421 anti-mouse CD150, PE-Cy7 anti-mouse Sca-1, BV786 anti-mouse c-Kit, APC anti-mouse CD16/32, PE anti-mouse CD34. Flow cytometry was performed on LSRFortessa (BD), and data were analyzed by using FlowJo software (Tree Star, Ashland, OR).

### Bone marrow transplantation (BMT)

BM cells were isolated from the tibias and femurs of diseased DKI mice and mice at the same age of the other groups including Dnmt3a^R878H/+^, Nras^G12D/+^ and WT mice (CD45.2^+^), respectively. BMT was then performed by injecting 4 × 10^5^ BM cells suspended in PBS into the tail vein of sublethally irradiated (350 cGy) recipient mice (CD45.1^+^) at 2–3 months old.

### Cell sorting

All the BM cells isolated from the various genotype mice were suspended in PBS buffer containing 1% FBS and subsequently stained with PE-conjugated Gr-1 antibody for half an hour. The cells were then washed and resuspended in 1–2 ml PBS buffer with 2% FBS. Gr-1^+^ cells were sorted by FACS ArialII (BD).

### RNA-seq

RNA was extracted from Gr-1^+^ cells using TRIzol-isopropanol precipitation. The quality of the RNA was checked using Nano drop, Qubit and Agilent 2100 Bioanalyzer. The mRNA of the qualified sample was enriched with mRNA Capture Beads, and then the fragmentation of mRNA was realized by the action of high temperature and metal ions. Using mRNA as template, a single chain cDNA was synthesized by six base random primers, followed by two strand cDNA synthesis reaction, then VAHTSTM DNA Clean Beads was used to purify double chain cDNA. The purified double strand cDNA was first repaired (poly A was added and sequenced), and VAHTSTM DNA Clean Beads was used to resize the fragment size. Finally, PCR amplification was carried out and the PCR products were purified by VAHTSTM DNA Clean Beads. The obtained library was then checked using Agilent High Sensitivity DNA Reagent with a 2100 Bioanalyzer, and 200-bp paired-end sequencing was carried out on an Illumina HiSeq.

### Quantitative real-time RT-PCR

The cDNA was synthesized used M-MLV reverse transcriptase (Invitrogen). RT-PCR was performed as described using reagents according to instructions of the manufacturer (Hieff TM qPCR SYBR® Green Master Mix; Yeasen). Reactions were performed on ABI PRISM 7500 Fast Real-Time PCR System or Applied Biosystems ViiA™ 7 Real-Time PCR System. Data were analyzed using formula 2^−ΔΔCt^.

### Western blot analysis

Total BM cells were lysed by RIPA Lysis Buffer (Beyotime). Protein assay was performed on Tecan infinite 200 Microplate Reader by BCA Protein Assay Kit (Beyotime). Antibodies were purchased from Cell Signaling Technology (anti-β-actin) and Abcam (anti-cMyc, anti-p62-cMyc).

### Statistical analysis

Kaplan-Meier survival analysis was performed and survival differences between groups were assessed with the Log-rank test, assuming significance at *P* < 0.05. Unpaired 2-tailed Student’s t-test was used to determine the significance between two data sets, assuming significance at *P* < 0.05.

## Results

### Dnmt3a R878H cooperates with Nras G12D to shorten the lifespan of DKI mice

**Fig. 1:**
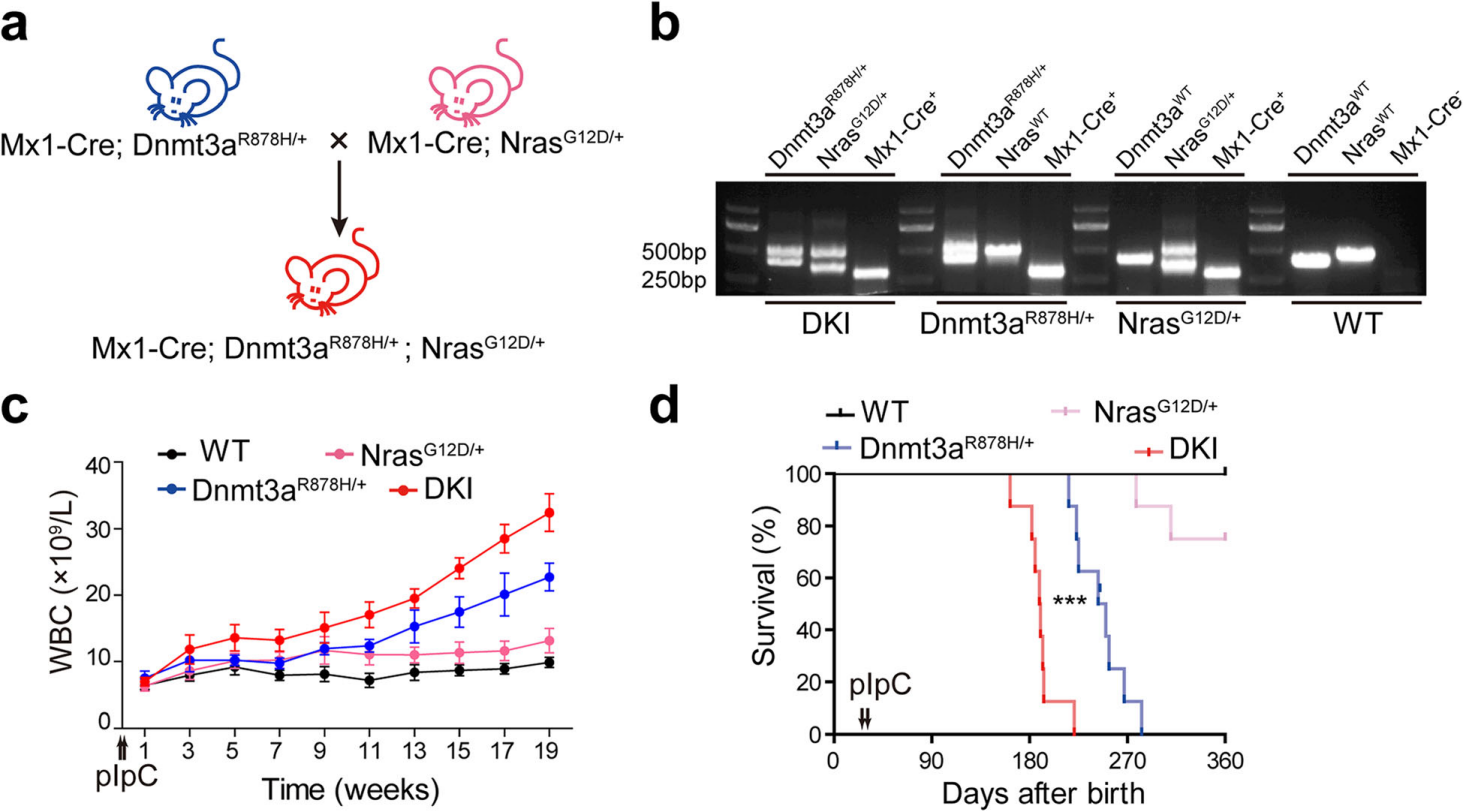
DKI mice show significantly shortened survival time. **a** Experimental scheme of the generation of conditional DKI mice. **b** Genotype identification of DKI mice (Mx1-Cre; Dnmt3a^R878H/+^; Nras^G12D/+^), Dnmt3a^R878H/+^ mice (Mx1-Cre; Dnmt3a^R878H/+^), Nras^G12D/+^ mice (Mx1-Cre; Nras^G12D/+^), and WT control. PCR was carried out by using the primers were listed in Additional file 1: Table S1. **c** Count of WBCs of mice with the indicated genotype after pIpC induction (*n* = 10 for each group). **d** Kaplan–Meier survival analysis of four groups of mice. (*n* = 8 for each group). ***P < 0.001

To discover the pathogenicity of *DNMT3A* and *NRAS* mutations in an endogenous expression environment, we crossed the Dnmt3a R878H KI mice (Dnmt3a^R878H/+^) with the Nras G12D KI mice (Nras^G12D/+^). The conditional KI mouse line Nras G12D and Dnmt3a R878H have been generated as previously described, respectively [13, 26, 27]. The DKI mouse model (Mx1-Cre; Dnmt3a^R878H/+^; Nras^G12D/+^) was established by crossing Mx1-Cre; Dnmt3a^R878H/+^ mice with Mx1-Cre; Nras^G12D/+^ mice (Fig. 1a). Their descendants’ genotypes were identified by PCR at genomic DNA level (Fig. 1b). PIpC was administered by intraperitoneal injection in KI and DKI mice to induce Cre-mediated gene expression. Nine weeks after pIpC induction, we found that white blood cells (WBCs) in PB of DKI mice began to significantly increase (Fig. 1c) compared with the Dnmt3a^R878H/+^ mice which also had gradually increased WBCs, whereas WBCs did not obviously changed in the Nras^G12D/+^ group, thus suggesting cooperation of *DNMT3A* and *NRAS* mutations could promote proliferative potential of hematopoietic cells. Interestingly, the DKI mice showed a significantly shortened survival (median survival time 189 days) compared with Dnmt3a^R878/+^ mice (median survival time 243 days) and Nras^G12D/+^ mice (Fig. 1d).

### Dnmt3a R878H cooperates with Nras G12D to induce a full-blown AML

**Fig. 2:**
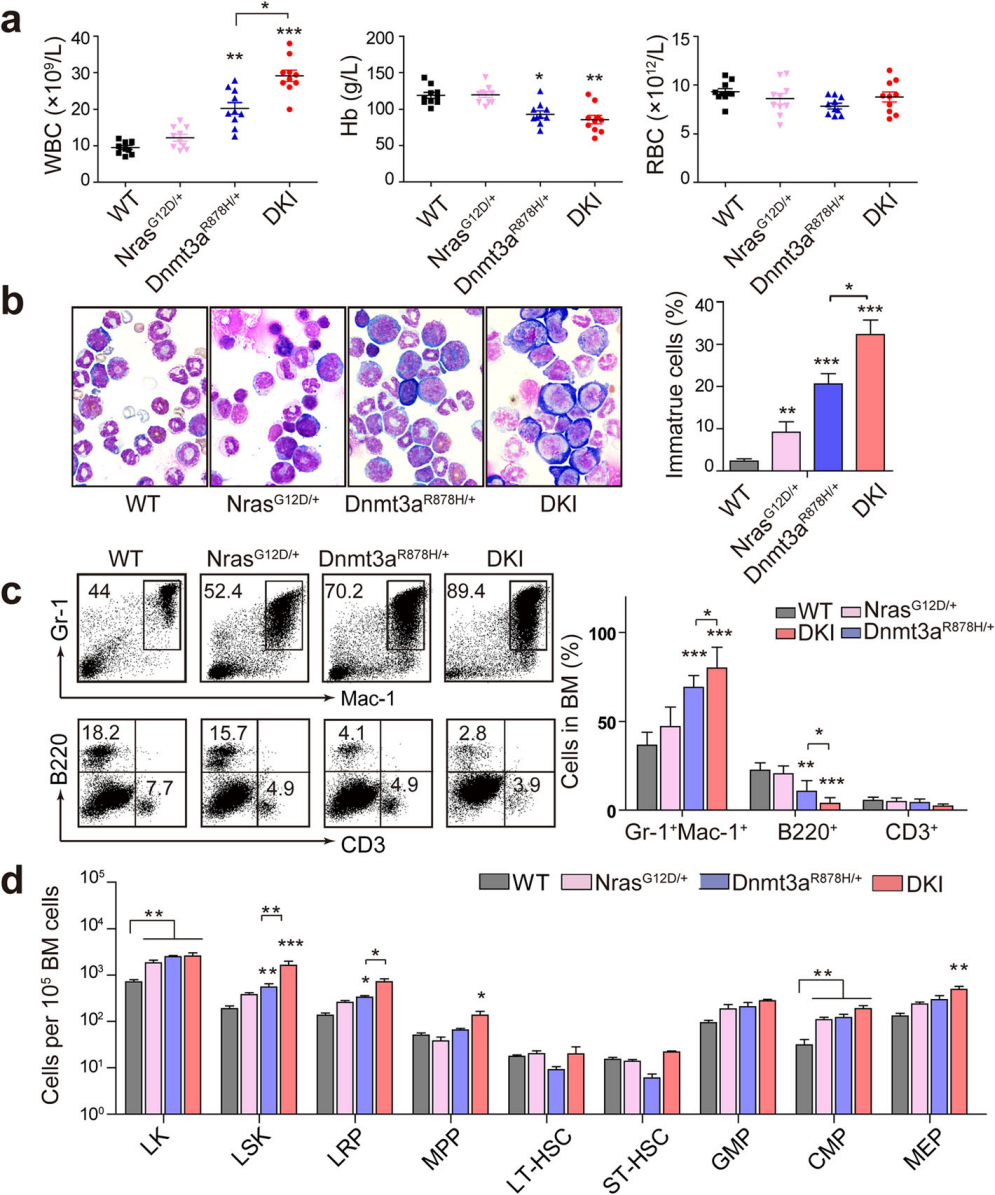
DKI mice develop a more severe AML compared with Dnmt3a^R878H/+^ mice. **a** Blood routine analysis of indicated mice 4 months after pIpC induction. **b** Morphological analysis of BM cells with Wright’s staining and quantification analysis of immature cells with a high nuclear/cytoplasmic ratio and fine chromatin in BM. **c** Flow cytometric analysis of BM cells from each group. Myeloid cells (Gr-1^+^ Mac-1^+^), B lymphocyte (B220^+^), T lymphocyte (CD3^+^). **d** Flow cytometric quantification of hematopoietic progenitor cell populations in BM of each group. LSK (Lineage^−^ Sca-1^+^ c-Kit^+^), LRP (CD150^−^ CD48^+^ LSK), MPP (CD150^−^ CD48^−^ LSK), LT-HSC (CD150^+^ CD48^−^ LSK), ST-HSC (CD150^+^ CD48^+^ LSK), GMP (CD34^+^ CD16/32^high^ LK), CMP (CD34^+^ CD16/32^med^ LK), MEP (CD34^−^ CD16/32^low^ LK). n = 10 for each group. Data were shown as Mean ± SEM values. * *P* < 0.05, *** *P* < 0.001, significance was determined by two-tailed Student’s t test

Routine blood test revealed elevated counts of WBCs, and reduced value of hemoglobin (Hb) and RBCs in Dnmt3a^R878H/+^ mice and DKI mice while Nras^G12D/+^ mice showed no obvious changes compared with wild-type (WT) 4 months after pIpC injection (Fig. 2a). Of note, DKI mice showed obviously increased amount of WBCs compared with Dnmt3a^R878H/+^ group (Fig. 2a). BM cells cytospin with Wright-Giemsa staining revealed higher proportion of immature cells in DKI mice (average, 32.5%) than in Dnmt3a^R878H/+^ mice (average, 20%) and Nras^G12D/+^ mice (average, 9.5%) (Fig. 2b). The flow cytometry analysis of BM cells showed an increase of myelomonocyte and decrease of lymphocyte with varying degrees in knock-in mice (Fig. 2c). The most extraordinary inversion of the proportion of myelomonocytes and lymphocytes was observed in DKI mice (Fig. 2c). DKI mice also showed significantly increased Lin^−^ Sca-1^+^ c-Kit^+^ (LSK) cells in contrast with Dnmt3a^R878H/+^ or Nras^G12D/+^ KI mice (Fig. 2d). Among the LSK cells, the quantity of lineage-restricted progenitors (LRPs) of both DKI and Dnmt3a^R878H/+^ mice remarkably expanded and DKI mice manifested more LRPs than Dnmt3a^R878H/+^ mice. And there was an obvious growth of multipotent progenitors (MPPs) in DKI mice whereas other groups remained unperturbed. We also observed some perturbation in the downstream progenitors of LSK cells with a notable raise of common myeloid progenitors (CMPs) in all the three groups of KI mice and a prominent increase of megakaryocyte-erythroid progenitors (MEPs) in DKI compared with WT mice (Fig. 2d). These data suggest that Dnmt3a R878H mutation with additional Nras G12D mutation could induce much more severe AML with distinct abnormal hematopoietic stem cells and progenitors.

### DKI mice develop more aggressive AML than Dnmt3a^R878H/+^ mice

**Fig. 3:**
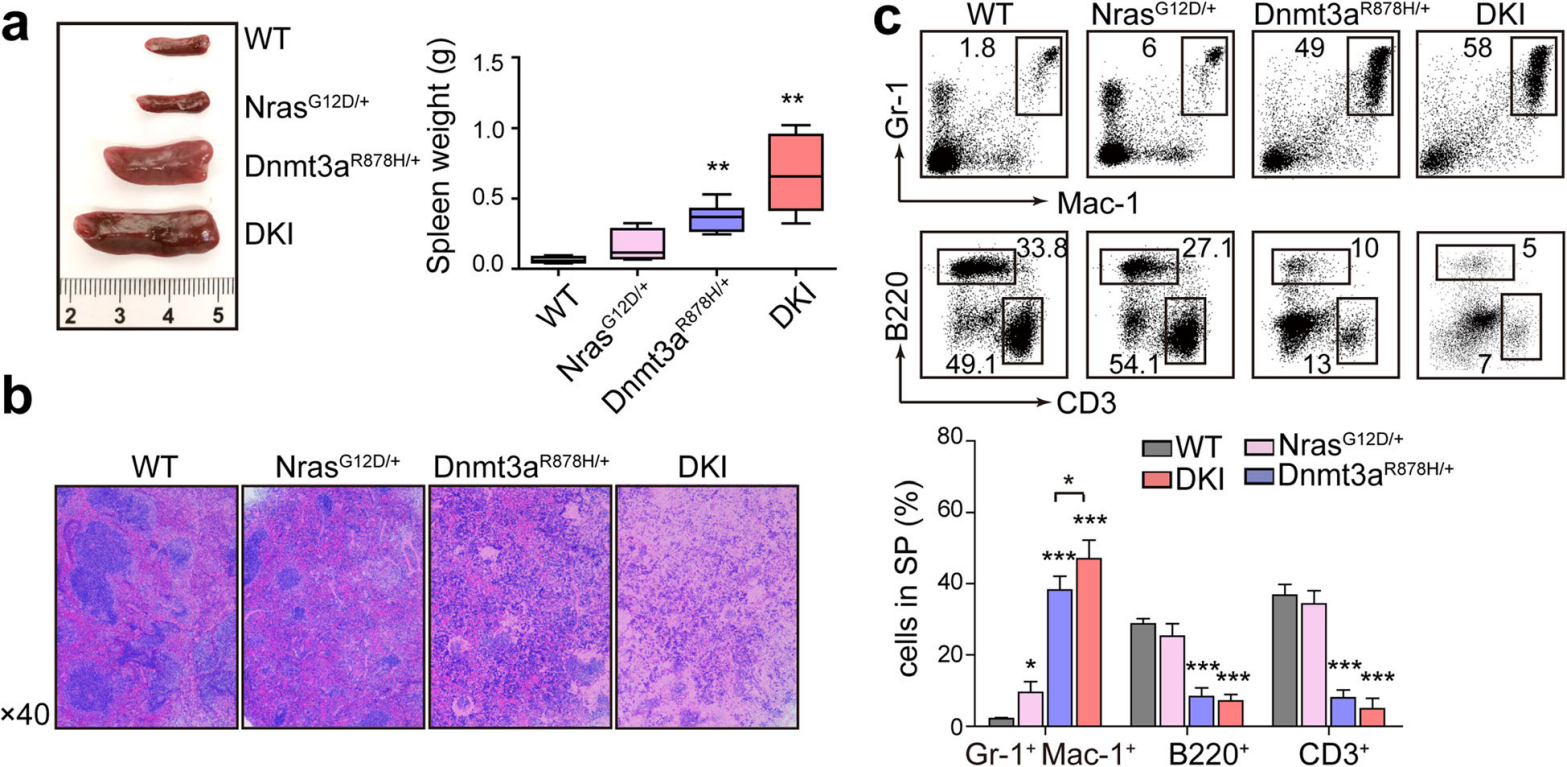
Dnmt3a R878H cooperates with Nras G12D to promote infiltration of leukemic cells. **a** Macroscopic appearance and weight of spleen. **b** Histological hematoxylin and eosin-stained histological sections of spleen from indicated mice. **c** Flow cytometric analysis of hematopoietic cells in spleen of each group. n = 10 for each group. Horizontal bars correspond to Mean ± SEM values. * *P* < 0.05, ** *P* < 0.01, ***P < 0.001, significance was determined by two-tailed Student’s t test

Both moribund DKI mice and Dnmt3a^R878H/+^ mice had distended abdomens, indicating characteristics of splenomegaly. Confirmed by the analysis of dissection, diseased DKI mice and Dnmt3a^R878H/+^ mice showed significant increased weight of spleen, while DKI mice displayed much heavier spleen compared with Dnmt3a^R878H/+^ mice (Fig. 3a). Pathological section revealed a more grievous effacement of splenic architecture as a result of extensive infiltration of leukemic cells in DKI mice in comparison to Dnmt3a^R878H/+^ mice and Nras^G12D/+^ mice (Fig. 3b). Confirmed by flow analysis of spleen cells, Gr-1^+^ Mac-1^+^ myeloid cells were significantly increased in DKI group comparison with Dnmt3a^R878H/+^ mice and Nras^G12D/+^ mice (Fig. 3c). These results showed that Dnmt3a R878H synergize with Nras G12D to transform cells into leukemic cells with a strong ability of infiltration.

### Leukemic cells from DKI mice show significant advantage of proliferation

**Fig. 4:**
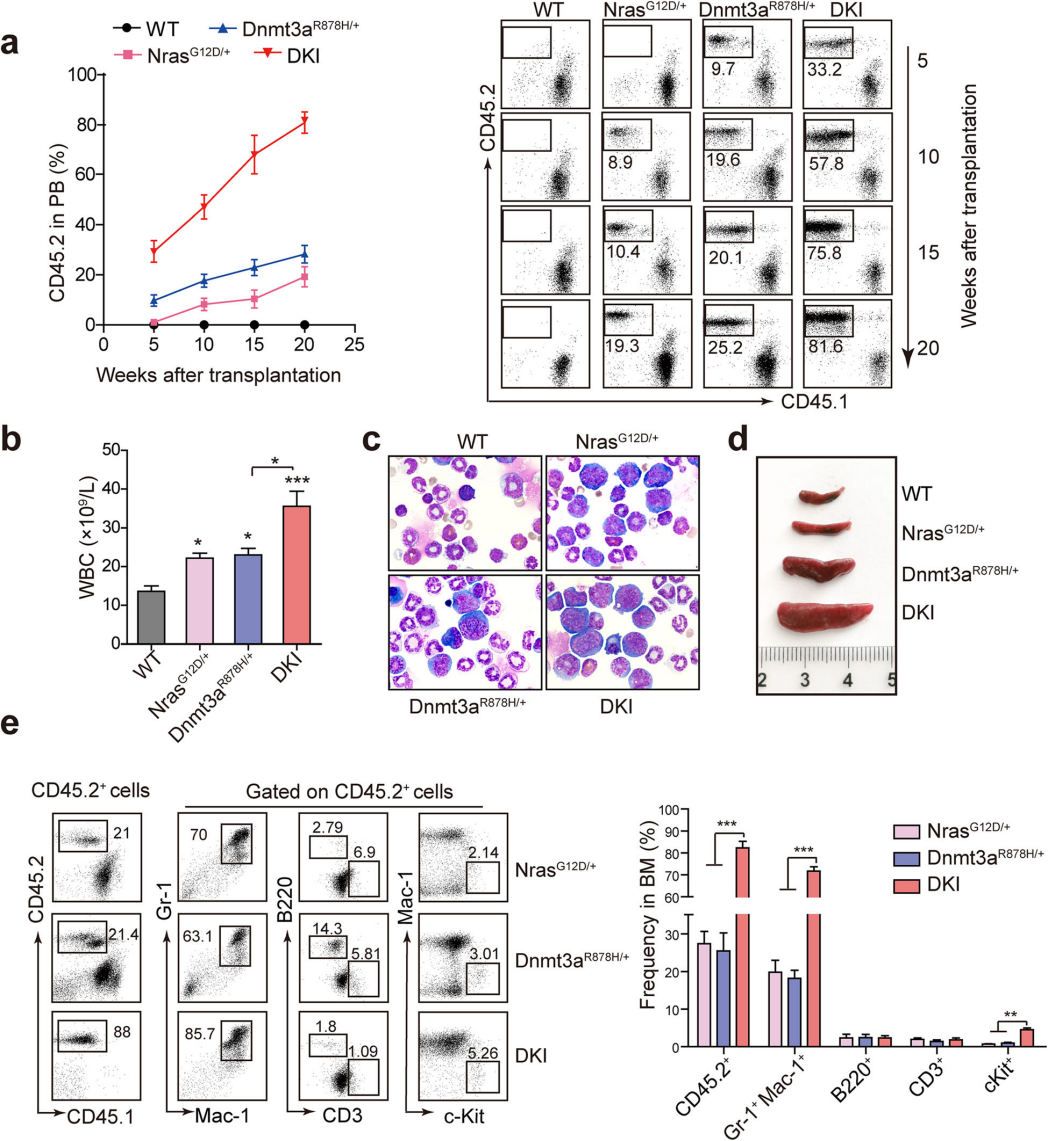
Dnmt3a R878H and Nras G12D-induced leukemia is transplantable with advantage of myeloid proliferation. **a** The percentage of CD45.2^+^ cells in PB of each group after BMT. (Left panel) Fold line diagram of the change of CD45.2^+^ cells. (Right panel) Representative flow cytometric results. **b** Count of WBCs in PB of each group 24 weeks after BMT. **c** Morphological analysis of BM cells with Wright’s staining of each group. **d** Macroscopic appearance of spleen of each group at the same time point. **e** Flow cytometric analysis of CD45.2^+^ cells and hematopoietic cell populations in BM of each group. CD45.2^+^ cells were gated for analysis of Gr-1^+^, B220^+^, CD3^+^ and c-Kit^+^ cells. *n* = 5 for each group. Horizontal bars correspond to Mean ± SEM values. * *P* < 0.05, ** *P* < 0.01, ***P < 0.001, significance was determined by two-tailed Student’s t test

To further investigate the cooperation between Dnmt3a R878H and Nras G12D, we performed secondary transplantation by transplanting freshly isolated BM cells from terminally diseased DKI mice and mice at the same age of Dnmt3a^R878H/+^, Nras^G12D/+^, and WT mice (with CD45.2 marker) into sublethally irradiated (350 cGy) recipient mice (with CD45.1 marker), respectively. The percentage of CD45.2^+^ cells in PB of recipient mice were then detected 5 weeks after transplantation by flow cytometry. There were almost no CD45.2^+^ cells detectable in the PB of Nras^G12D/+^ and WT mice during the first 5 weeks post transplantation, while Dnmt3a^R878H/+^ mice showed an average of 13.7% CD45.2^+^ cells. In contrast, recipient mice of DKI showed an average of 30% CD45.2^+^ cells (Fig. 4a). During the next 15 weeks, CD45.2^+^ cells in PB from DKI mice continuously grew to an average of 80.6%, whereas there was only a moderate increase of CD45.2^+^ cells from mice with single mutation (an average of 28.2% for Dnmt3a^R878H/+^ and 19.2% for Nras^G12D/+^) (Fig. 4a). These results revealed that cooperation of Dnmt3a R878H and Nras G12D could synergistically promote the proliferation of transformed hematopoietic cells. Phenotype analysis at 24 weeks after transplantation showed that the recipient mice of DKI developed an AML phenotype similar to that observed in primary mice. The recipient mice harboring two kind of mutations showed a significant increase of WBCs compared with Dnmt3a R878H or Nras G12D mutation alone (Fig. 4b). Compared to the single mutation recipient mice, mice with double mutations displayed much more immature cells in BM and significantly enlarged spleen (Fig. 4c and Fig. 4d). Flow cytometry analysis of CD45.2^+^ cells in the BM revealed a remarkable altered granulocyte/lymphocyte ratio and increased c-Kit^+^ cells in DKI recipient mice (Fig. 4e). These data suggest that cooperation of Dnmt3a R878H with additional Nras G12D mutation could accelerate the onset of AML and worsen disease progression.

### Cooperation of Dnmt3a R878H and Nras G12D causes transcriptional alteration

**Fig. 5:**
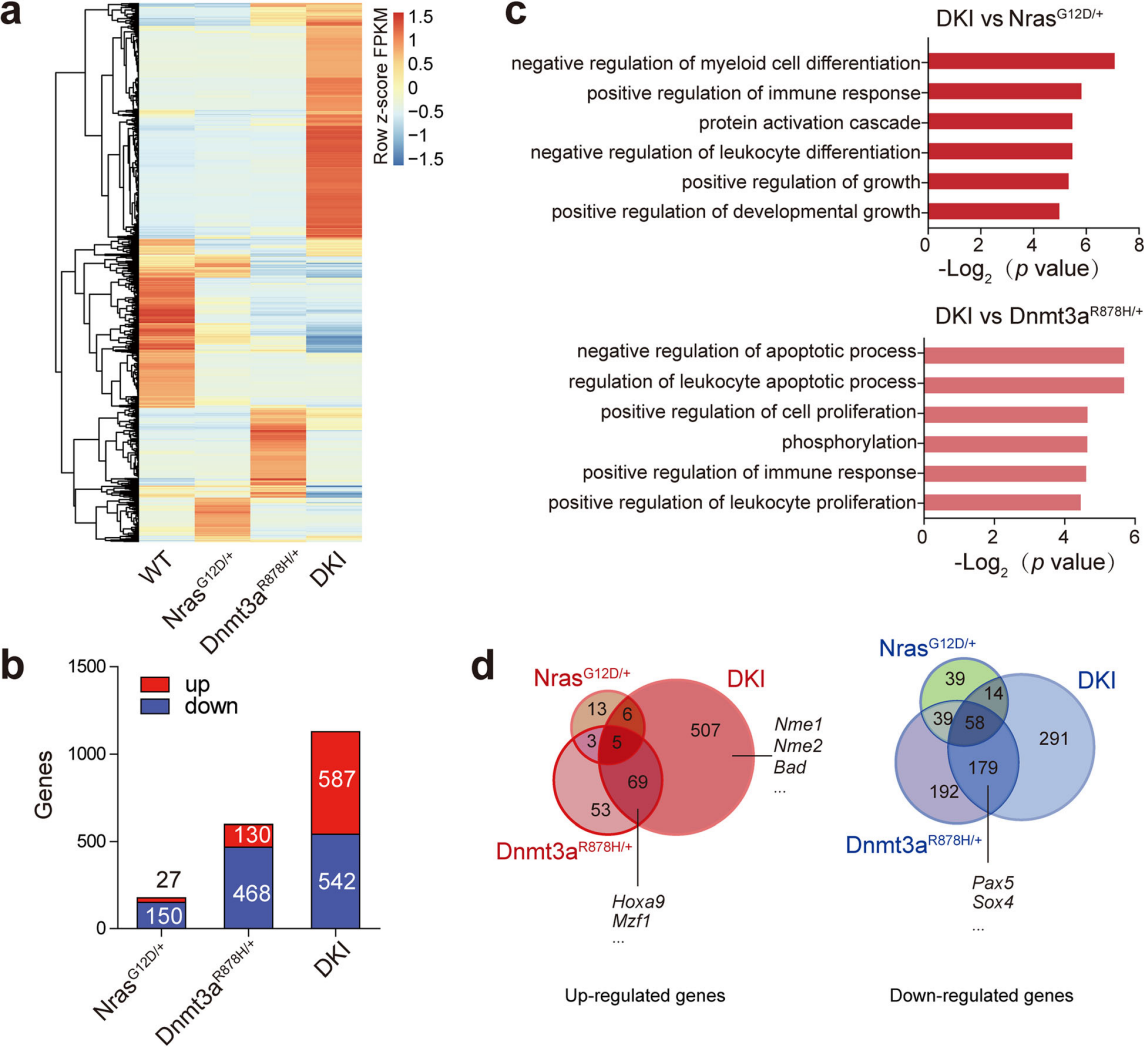
GO enrichment analysis and Venn analysis of dysregulated genes. **a** Heat map of differentially expressed genes in each mutant genotype group compared with WT mice. **b** Up-regulated and down-regulated genes in each mutant genotype group compared with WT mice. **c** GO (DAVID) analysis of pairwise comparisons of up-regulated genes in DKI mice compared with Nras^G12D/+^ (upper panel) and Dnmt3a^R878H/+^ (lower panel) mice, respectively. **d** Venn diagram of genes up-regulated (left panel) and down-regulated (right panel) in KI and DKI mice in comparison to WT. |log2Ratio| ≥ 1, false discovery rate (FDR), q-value ≤0.05. Genes with significant change in DKI mice were showed in Additional file 2: Table S2

To clarify the mechanisms underlying the cooperation of Dnmt3a R878H and Nras G12D in leukemogenesis, we performed transcriptome profiling by RNA-seq of Gr-1^+^ BM cells harvested from diseased DKI mice as well as Dnmt3a^R878H/+^, Nras^G12D/+^, and WT mice at the same age. The Gr-1^+^ cells were sorted for RNA-seq due to an observation of distinct change of Gr-1^+^ cells in both DKI and Dnmt3a R878H induced AML mice which is consistent with our previous report [13]. There were more differentially expressed genes in DKI mice compared with Dnmt3a^R878H/+^ and Nras^G12D/+^ mice (Fig. 5a). In comparison to WT, DKI displayed 1129 significant differential genes, while Dnmt3a^R878H/+^ and Nras^G12D/+^ groups had 598 and 177 significant differential genes, respectively (Fig. 5b). Notably, 587 genes in DKI, 130 genes in Dnmt3a^R878H/+^and 27 genes in Nras^G12D/+^ mice were up-regulated compared with WT (Fig. 5b). Gene ontology (GO) analysis revealed that the up-regulated genes in DKI mice compared with Nras^G12D/+^ mice were mainly related to positive of regulation of growth, negative regulation of differentiation, immune response and protein activation cascade (Fig. 5c upper panel). And the upregulated genes in DKI mice compared with Dnmt3a^R878H/+^ were mainly related to positive regulation of proliferation, negative regulation of apoptotic process, phosphorylation and immune response (Fig. 5c lower panel). Of note, the cooperation of *Dnmt3a* mutation and *Nras* mutation caused distinct dysregulated genes compared with *Dnmt3a* or *Nras* mutation alone. Venn analysis of differential genes also showed a dramatic amount of upregulated genes in DKI mice (Fig. 5d), including transcription factors *Nme1* and *Nme2*, which were shown as potential molecular markers associated with prognosis in some studies [28, 29], and *Bad* gene involved in apoptotic process whose expression positively correlates with the disease stage [30]. In addition, *Hoxa9* and *Mzf1* involved in development of myeloid leukemia were up-regulated [31, 32], while genes associated with differentiation, including *Pax5* and *Sox4*, were down-regulated in both DKI and Dnmt3a^R878H/+^ groups [33, 34] (Fig. 5d). Of note, the cooperation of *Dnmt3a* mutation and *Nras* mutation caused some newly dysregulated genes compared with the single mutation alone. These results indicate that Dnmt3a R878H plays a key role in regulating proliferation and differentiation, whereas the acquisition of Nras G12D provide an additional proliferation signal and negative regulation of apoptosis, thus contributing to promote leukemogenesis.

### Cooperation of Dnmt3a R878H and Nras G12D activates Myc pathway

**Fig. 6:**
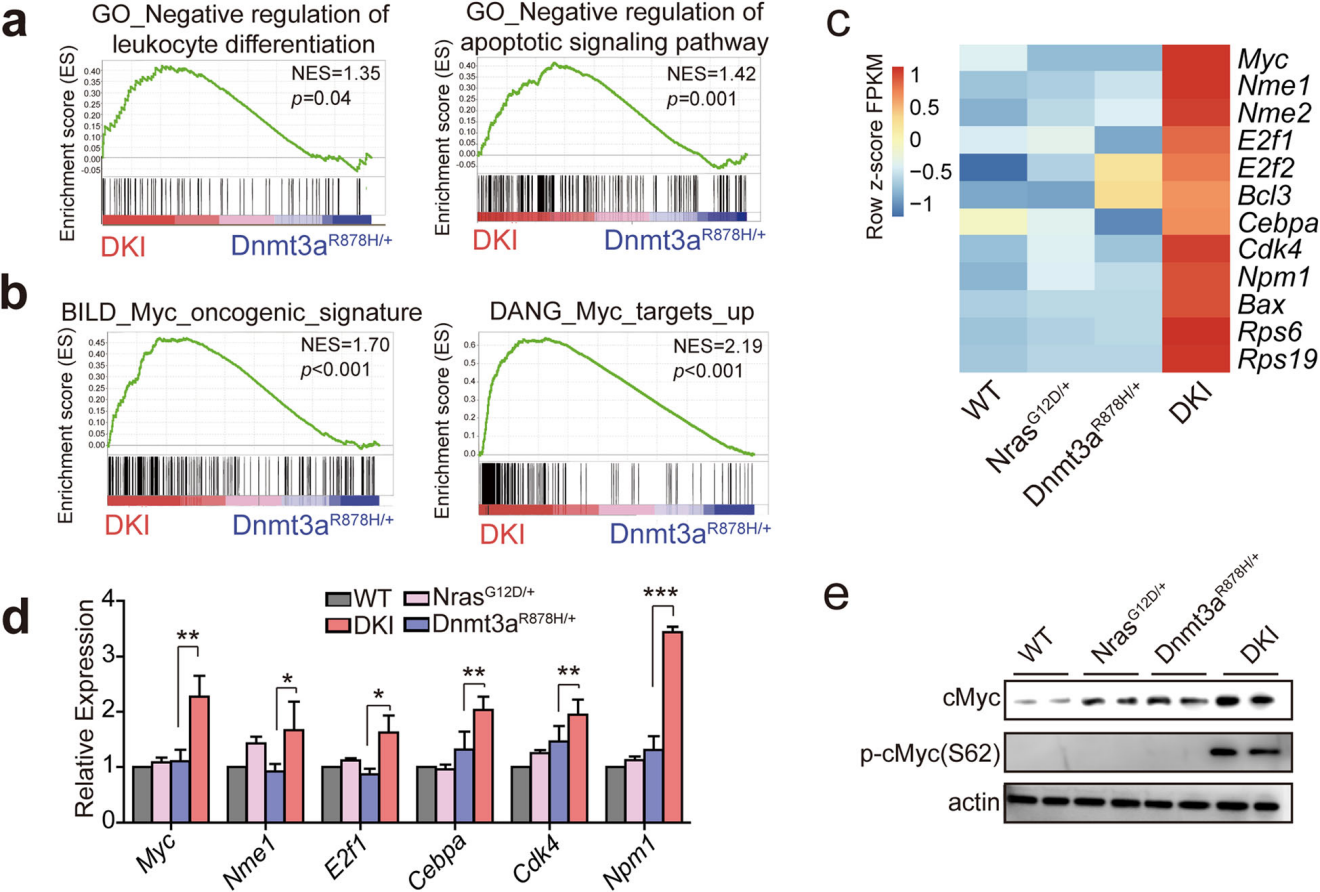
The activation of Myc oncogenic signature in DKI mice. **a** GSEA shows enrichment of differentiation and apoptosis gene sets in DKI groups compared with Dnmt3a^R878H/+^ group. **b** GSEA shows enrichment of *Myc* oncogenic signature and Myc target gene sets in DKI group compared with Dnmt3a^R878H/+^ group. NES, normalized enrichment score. **c** Heat map of up-regulated *Myc* and Myc target genes in DKI mice compared with WT, Dnmt3a^R878H/+^ and Nras^G12D/+^ mice. **d** The gene expression levels were validated by real-time RT-PCR. Primers were listed in Additional file 3: Table S3. **e** Western blot analysis of cMyc and p62-cMyc. Horizontal bars correspond to Mean ± SEM values. * *P* < 0.05, ** *P* < 0.01, ***P < 0.001, significance was determined by two-tailed Student’s t test

To further investigate the key upregulated genes in the pathogenesis of DKI mice, we performed gene set enrichment analysis (GSEA) of RNA-seq data. GSEA showed a significant enrichment of the gene sets related with negative regulation of differentiation and apoptotic signaling in the Gr-1^+^ cells of DKI groups compared with Dnmt3a^R878H/+^ mice (Fig. 6a). Interestingly, gene sets associated with Myc oncogenic signature and Myc targets were significantly enriched in DKI groups (Fig. 6b). The heat map displayed the dysregulated genes correspond to gene sets significantly affected according to GSEA (Fig. 6c). Compared with Dnmt3a^R878H/+^ mice and Nras^G12D/+^ mice, DKI mice showed obvious up-regulation of transcription factor *Myc* and a serial of Myc target genes [35], including *Nme* family genes, *Cebpa*, *Npm1* and *Bcl3* genes involved in the development of leukemia [36–38], *Cdk4* and *Bax* genes related with cell cycle and apoptosis process [39, 40]. The upregulated-genes were validated by real-time qPCR (Fig. 6 d). The oncogene c*Myc* is one of the most common genetic alterations in human cancers [41]. *cMyc* has been reported to be implicated in regulating a wide variety of biological activities, and particularly in balancing the self-renewal and differentiation of hematopoietic stem cell [42, 43]. Western blot showed that the expression of cMyc protein was increased in DKI mice compared with Dnmt3a^R878H/+^ or Nras^G12D/+^ single KI mice, and cMyc s62 phosphorylation, which was reported to increase cMyc protein stability and activity [44, 45], was also remarkably overexpressed in DKI mice. Therefore, up-regulation of *Myc* and Myc target genes related to proliferation and apoptosis may contribute to promote the process of *Dnmt3a* mutation-induced AML.

## Discussion

As one of common epigenetic alterations in AML, *DNMT3A* mutation has been demonstrated to play an important role in the pathogenesis of leukemia. Many studies have suggested that mutant *DNMT3A* requires additional gene mutations to cause full-blown AML. In this regard, a previous report showed the cooperation of *DNMT3A* mutation and *RAS* mutation in leukemogenesis by using retroviral transduced mouse model [12]. However, the conditional KI approach is better than retroviral transduction system to recapitulate human leukemic features in mice. In this study, we created the first conditional DKI mouse model expressing Dnmt3a R878H and Nras G12D under the control of endogenous promoters by crossing Dnmt3a^R878H/+^ KI mice and Nras^G12D/+^ KI mice. By generating the conditional DKI mouse model, we discovered that cooperation of Dnmt3a R878H and Nras G12D could lead to a much earlier onset and more severe AML characterized by significantly increased WBCs, elevated immature cells, splenomegaly and shortened survival time, compared with Dnmt3a^R878H/+^ or Nras^G12D/+^ KI mice. In this pilot study, preliminary data derived from 10 mice per group were statistically analyzed.

Dnmt3a^R878H/+^ mice have been reported to develop moderate AML with considerably increased hematopoietic stem and progenitor cells especially LSK cells which were demonstrated to harbor leukemia-initiating cells [13]. Here, we showed that the LSK compartment was significantly enlarged in DKI AML mice compared with the diseased Dnmt3a^R878H/+^ mice, suggesting that *DNMT3A* mutation with acquisition of additional genetic abnormality such as *NRAS* mutation could significantly promote the leukemogenic transformation and proliferation of hematopoietic cells.

We investigated the mechanism underlying the leukemic phenotype induced by the cooperation of Dnmt3a R878H and Nras G12D. DKI mice showed much more up-regulated genes contributing to positive regulation of growth, negative regulation of differentiation and negative regulation of apoptosis process than Dnmt3a^R878H/+^ KI mice and Nras^G12D/+^ KI mice. Interestingly, the genes associated with phosphorylation and protein activation cascade were also up-regulated in DKI mice, indicating abnormal phosphorylation and protein activation caused by the cooperation of *Dnmt3a* and *Nras* mutation. We found that *Dnmt3a* mutation together with *Nras* mutation could not only increase the expression of *Myc* oncogene and Myc target genes, but also could induce the s62 phosphorylation of cMyc which has been reported to play a key role in increasing the protein stability and activating the Myc oncogenic signature [44, 45]. Therefore, the activation of Myc pathway may contribute to the pathogenesis of full-blown AML caused by the cooperation of *Dnmt3a* mutation and *Nras* mutation.

In this work, we generated a novel DKI mouse model to investigate the function and mechanism of *Dnmt3a* mutation under the presence of *Nras* mutation in inducing a full-blown AML. We discovered that *Dnmt3a* mutation and *Nras* mutation could cooperate to induce a much more severe AML than *Dnmt3a* or *Nras* mutation alone. In the mechanism study, we revealed that DKI mice showed significantly altered gene expression patterns with involvement of Myc pathway activation, indicating a potential therapeutic target in *DNMT3A* mutation-related leukemia which needs to be further investigated.

## Conclusions

In conclusion, we discovered that cooperation of *Dnmt3a* mutation with *Nras* mutation could synergistically induce AML under the control of endogenous promoter/enhancer in mice, and activation of Myc pathway is one of the key players in the disease mechanism. Our study thus provides a unique mouse model that recapitulates many aspects of human AML and a potential therapeutic target for *DNMT3A* mutation-related leukemia.

## Supplementary information

**Additional file 1: Table S1.** Primers for identifying genotypes of Knock-in mice.

**Additional file 2: Table S2.** Genes with Different Expression Patterns of Gr-1 Positive Cells Between DKI and WT Mice.

**Additional file 3: Table S3.** Primers for validating the results of RNA-seq analysis.

## Data Availability

All data generated or analyzed during this study are included in this published article.

## Abbreviations

AML: Acute myeloid leukemia
BM: Bone marrow
BMT: Bone marrow transplantation
CMPs: Common myeloid progenitors
DKI: Double knock-in
DNMT3A: DNA methytransferase 3A
FBS: fetal bovine serum
Hb: Hemoglobin
HSCs: Hematopoietic stem cells
KI: Knock-in
LRPs: Lineage-restricted progenitors
LSKs: Lin^−^ Sca-1^+^ c-Kit^+^ cells
MEPs: Megakaryocyte-erythroid progenitors
MPPs: Multipotent progenitors
PB: Peripheral blood
pIpC: Polyinosinic-polycytidylic acid
RBCs: Red blood cells
RNA-seq: RNA-sequencing
WBCs: White blood cells
WT: Wild type
